# (*E*)-2-[(2,4,6-Tri­meth­oxy­benzyl­idene)amino]­phenol^1^

**DOI:** 10.1107/S1600536813032996

**Published:** 2013-12-14

**Authors:** Narissara Kaewmanee, Suchada Chantrapromma, Nawong Boonnak, Ching Kheng Quah, Hoong-Kun Fun

**Affiliations:** a Department of Chemistry and Center of Excellence for Innovation in Chemistry, Faculty of Science, Prince of Songkla University, Hat-Yai, Songkhla 90112, Thailand; b Department of Chemistry, Faculty of Science, Prince of Songkla University, Hat-Yai, Songkhla 90112, Thailand; c Faculty of Traditional Thai Medicine, Prince of Songkla University, Hat-Yai, Songkhla 90112, Thailand; d X-ray Crystallography Unit, School of Physics, Universiti Sains Malaysia, 11800 USM, Penang, Malaysia; e Department of Pharmaceutical Chemistry, College of Pharmacy, King Saud University, PO Box 2457, Riyadh 11451, Saudi Arabia

## Abstract

There are two independent mol­ecules in the asymmetric unit of the title compound, C_16_H_17_NO_4_, with similar conformations but some differences in their bond angles. Each mol­ecule adopts a *trans* configuration with respect to the methyl­idene C=N bond and is twisted with a dihedral angle between the two substituted benzene rings of 80.52 (7)° in one mol­ecule and 83.53 (7)° in the other. All meth­oxy groups are approximately coplanar with the attached benzene rings, with C_meth­yl_—O—C—C torsion angles ranging from −6.7 (2) to 5.07 (19)°. In the crystal, independent mol­ecules are linked together by O—H⋯N and O—H⋯O hydrogen bonds and a π–π inter­action [centroid–centroid distance of 3.6030 (9) Å], forming a dimer. The dimers are further linked by weak C—H⋯O inter­actions and another π–π inter­action [centroid–centroid distance of 3.9452 (9) Å] into layers lying parallel to the *ab* plane.

## Related literature   

For organic bond-length data, see: Allen *et al.* (1987 ▶). For related literature on hydrogen-bond motifs, see: Bernstein *et al.* (1995 ▶). For background to and application of aza-stilbene, see: Cheng *et al.* (2010 ▶); da Silva *et al.* (2011 ▶); Fujita *et al.* (2012 ▶); Lu *et al.* (2012 ▶); Tamizh *et al.* (2012 ▶). For related structures, see: Kaewmanee *et al.* (2013 ▶); Sun *et al.* (2011 ▶).
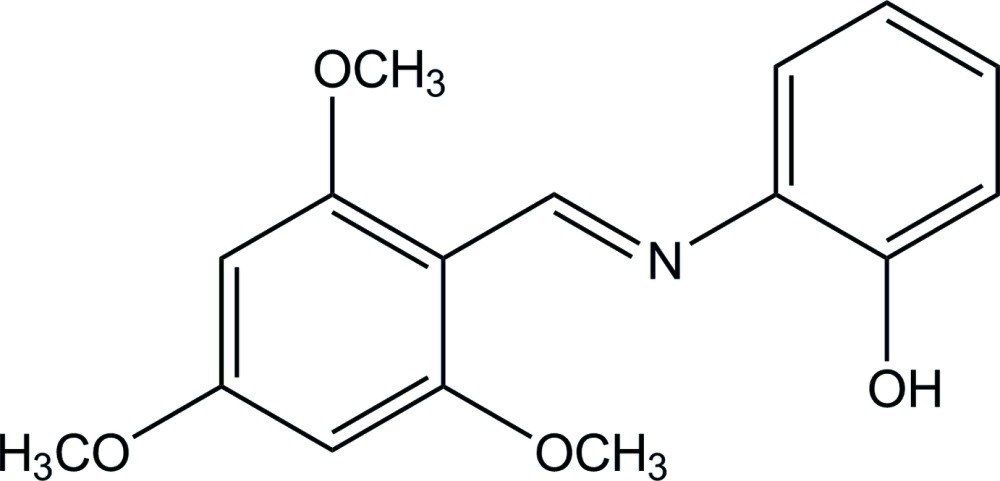


## Experimental   

### 

#### Crystal data  

C_16_H_17_NO_4_
*M* *_r_* = 287.31
Triclinic, 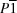

*a* = 7.3819 (6) Å
*b* = 11.7036 (9) Å
*c* = 16.4373 (13) Å
α = 89.469 (2)°
β = 85.616 (2)°
γ = 80.456 (2)°
*V* = 1396.34 (19) Å^3^
*Z* = 4
Mo *K*α radiation
μ = 0.10 mm^−1^
*T* = 100 K
0.49 × 0.16 × 0.16 mm

#### Data collection  

Bruker SMART APEX2 CCD area-detector diffractometer
Absorption correction: multi-scan (*SADABS*; Bruker, 2009 ▶) *T*_min_ = 0.954, *T*_max_ = 0.985
29313 measured reflections
8129 independent reflections
5872 reflections with *I* > 2σ(*I*)
*R*_int_ = 0.048

#### Refinement  

*R*[*F* ^2^ > 2σ(*F* ^2^)] = 0.052
*wR*(*F* ^2^) = 0.148
*S* = 1.02
8129 reflections
393 parameters
H atoms treated by a mixture of independent and constrained refinement
Δρ_max_ = 0.41 e Å^−3^
Δρ_min_ = −0.32 e Å^−3^

### 

Data collection: *APEX2* (Bruker, 2009 ▶); cell refinement: *SAINT* (Bruker, 2009 ▶); data reduction: *SAINT*; program(s) used to solve structure: *SHELXTL* (Sheldrick, 2008 ▶); program(s) used to refine structure: *SHELXTL*; molecular graphics: *SHELXTL*; software used to prepare material for publication: *SHELXTL*, *PLATON* (Spek, 2009 ▶), *Mercury* (Macrae *et al.*, 2006 ▶) and *publCIF* (Westrip, 2010 ▶).

## Supplementary Material

Crystal structure: contains datablock(s) global, I. DOI: 10.1107/S1600536813032996/is5324sup1.cif

Structure factors: contains datablock(s) I. DOI: 10.1107/S1600536813032996/is5324Isup2.hkl

Supporting information file. DOI: 10.1107/S1600536813032996/is5324Isup3.cml

Additional supporting information:  crystallographic information; 3D view; checkCIF report

## Figures and Tables

**Table 1:** Hydrogen-bond geometry (Å, °)

*D*—H⋯*A*	*D*—H	H⋯*A*	*D*⋯*A*	*D*—H⋯*A*
O4*A*—H1*O*4⋯O3*B*	0.88 (2)	2.53 (2)	2.9924 (15)	114.0 (17)
O4*A*—H1*O*4⋯N1*B*	0.88 (2)	1.96 (2)	2.7897 (15)	158 (2)
O4*B*—H2*O*4⋯N1*A*	0.88 (2)	2.00 (2)	2.8013 (16)	151 (2)
O4*B*—H2*O*4⋯N1*B*	0.88 (2)	2.35 (2)	2.7891 (16)	111.1 (18)
C13*B*—H13*B*⋯O3*A* ^i^	0.95	2.59	3.4639 (19)	154
C15*B*—H15*D*⋯O4*A* ^ii^	0.98	2.43	3.3847 (18)	164

Symmetry codes: (i) 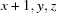; (ii) 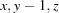.

**Fig. 1.**
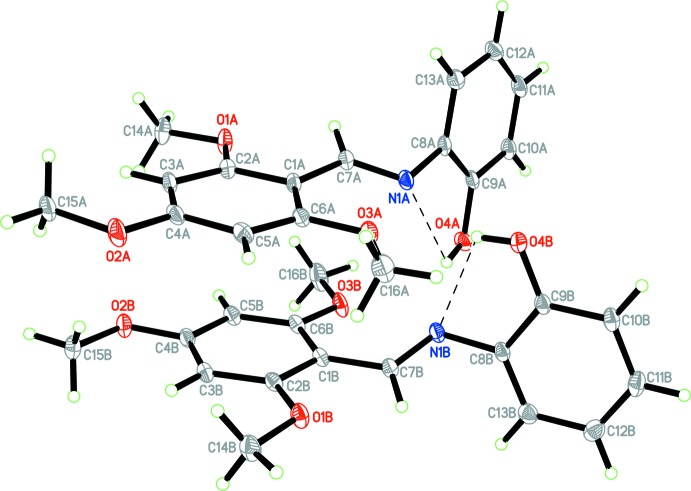
The asymmetric unit of the title compound showing 40% probability displacement ellipsoids and the atom-numbering scheme. Intramolecular hydrogen bonds are drawn as dashed lines.

**Fig. 2.**
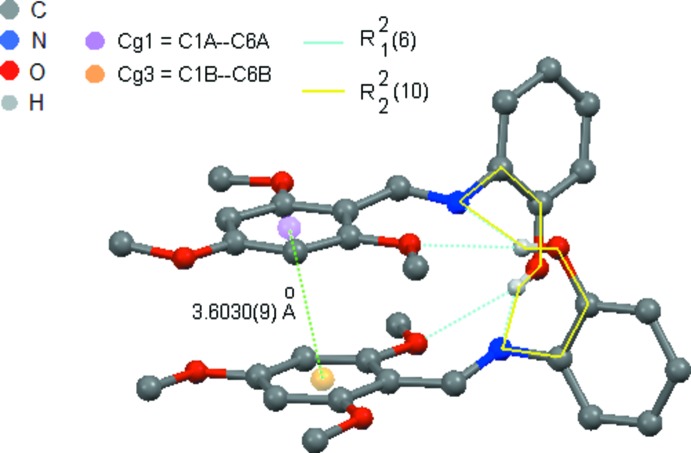
R21(6) and R21(10) ring motifs and a π–π interaction in the crystal of the title compound.

**Fig. 3.**
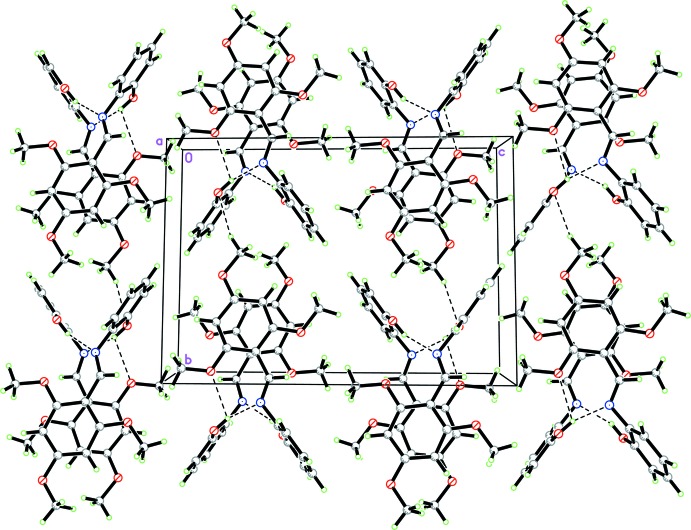
The crystal packing of the title compound viewed along the a axis. Hydrogen bonds are drawn as dashed lines.

## supplementary crystallographic information

### 1. Comment

Aza-stilbenes are a special group of compounds in the Schiff base family which can be synthesized by the reaction of aldehyde with aniline. Aza-stilbenes have been shown to possess potent biological properties such as antibacterial (Tamizh *et al.*, 2012), antioxidant (Cheng *et al.*, 2010; Lu *et al.*, 2012), antifungal (da Silva *et al.*, 2011) and antiproliferative (Fujita *et al.*, 2012) activities. The interesting biological activities of aza-stilbenes have led us to synthesize the title compound (I) and study its antibacterial and antioxidant activities. Our antibacterial assay have shown that (I) possesses moderate to weak antibacterial activity against *B. subtilis*, *S. aureus*, *P. aeruginosa*, *S. typhi* and *S.sonnei* with the MIC values in the range of 37.5 to 150 µg/ml. In addition (I) also shows interesting antioxidant activity by DPPH assay with the IC_50_ value of 0.080 ± 0.0004 µg/ml. We report here the crystal structure of the title compound.

There are two independent molecules, *A* and *B* in the asymmetric unit of the title compound, with similar conformations but some differences in bond angles (Fig. 1). The molecular structure exists in a *trans* configuration with respect to the methylidene C7═N1 double bond [1.2868 (13) and 1.2823 (19) Å for molecules *A* and *B*, respectively] and with the torsion angle C8—N1—C7—C1 = -175.58 (13)° for molecule *A* [177.48 (13)° for molecule *B*]. The molecule is twisted with the dihedral angle between the two substituted benzene rings being 80.52 (7)° in molecule *A* and 83.53 (7)° in molecule *B*. In both molecules, the three methoxy groups are co-planar with their bound benzene rings with the C14—O1—C2—C3 = -3.2 (2)°, C15—O2—C4—C3 = -6.7 (2)° and C16—O3—C6—C5 = -1.5 (2)° in molecule *A*, and the corresponding values are 5.07 (19), 1.86 (19) and -1.7 (2)° in molecule *B*. In each molecule, an intramolecular O—H···N hydrogen bond (Fig. 1 and Table 1) generates an S(5) ring motif (Bernstein *et al.*, 1995). The bond distances are in normal ranges (Allen *et al.*, 1987) and are comparable with the related structures (Kaewmanee *et al.*, 2013; Sun *et al.*, 2011).

In the crystal structure, the molecules are linked into dimers by O—H···N and O—H···O hydrogen bonds (Table 1) which form two *R*^2^_1_(6) ring motifs and an *R*^2^_2_(10) ring motif (Fig. 2). These dimers are further linked by C—H···O interactions (Table1) into chains along the *b* direction which arranged into sheets parallel to the *ab* plane (Fig. 3). There are π–π interactions with *Cg*1···*Cg*3 and *Cg*1···*Cg*3^iv^ distances of 3.6030 (9) and 3.9452 (9) Å, respectively (Fig. 2) [symmetry code: (iv) = -1 + *x*, *y*, *z*]; *Cg*1 and *Cg*3 are the centroids of C1A–C6A and C1B–C6B rings, respectively.]

### 2. Experimental

The title compound (I) was prepared by mixing 1:1 molar ratio solutions of 2-aminophenol (2.5 mmol, 0.25 g) in toluene (20 ml) and 2,4,6-trimethoxybenzaldehyde (2.5 mmol, 0.50 g) in toluene (20 ml). The reaction mixture was refluxed for around 4 h, yielding white solids , which was collected by filtration, washed with cold ethanol and dried in air. Colorless block-shaped single crystals suitable for *X*-ray structure determination were recrystalized from methanol by slow evaporation of the solvent at room temperature after several days (m.p. 450–452 K).

### 3. Refinement

Hydroxy H atom was located in a difference map and refined freely. The remaining H atoms were fixed geometrically and allowed to ride on their parent atoms, with d(C—H) = 0.95 Å for aromatic and CH, and 0.98 Å for CH_3_ atoms. The *U*_iso_(H) values were constrained to be 1.5*U*_eq_ of the carrier atom for water and methyl H atoms and 1.2*U*_eq_ for the remaining H atoms. A rotating group model was used for the methyl groups.

### Crystal data

**Table:**

C_16_H_17_NO_4_	*Z* = 4
*M**_r_* = 287.31	*F*(000) = 608
Triclinic, *P*1	*D*_x_ = 1.367 Mg m^−^^3^
Hall symbol: -P 1	Melting point = 450–452 K
*a* = 7.3819 (6) Å	Mo *K*α radiation, λ = 0.71073 Å
*b* = 11.7036 (9) Å	Cell parameters from 8129 reflections
*c* = 16.4373 (13) Å	θ = 2.5–30.0°
α = 89.469 (2)°	µ = 0.10 mm^−^^1^
β = 85.616 (2)°	*T* = 100 K
γ = 80.456 (2)°	Block, colourless
*V* = 1396.34 (19) Å^3^	0.49 × 0.16 × 0.16 mm

### Data collection

**Table:**

Bruker SMART APEX2 CCD area-detector diffractometer	8129 independent reflections
Radiation source: fine-focus sealed tube	5872 reflections with *I* > 2σ(*I*)
Graphite monochromator	*R*_int_ = 0.048
Detector resolution: 8.33 pixels mm^-1^	θ_max_ = 30.0°, θ_min_ = 2.5°
ω scans	*h* = −10→10
Absorption correction: multi-scan (*SADABS*; Bruker, 2009)	*k* = −16→16
*T*_min_ = 0.954, *T*_max_ = 0.985	*l* = −23→23
29313 measured reflections	

### Refinement

**Table:**

Refinement on *F*^2^	Primary atom site location: structure-invariant direct methods
Least-squares matrix: full	Secondary atom site location: difference Fourier map
*R*[*F*^2^ > 2σ(*F*^2^)] = 0.052	Hydrogen site location: inferred from neighbouring sites
*wR*(*F*^2^) = 0.148	H atoms treated by a mixture of independent and constrained refinement
*S* = 1.02	*w* = 1/[σ^2^(*F*_o_^2^) + (0.0788*P*)^2^ + 0.2397*P*] where *P* = (*F*_o_^2^ + 2*F*_c_^2^)/3
8129 reflections	(Δ/σ)_max_ = 0.001
393 parameters	Δρ_max_ = 0.41 e Å^−^^3^
0 restraints	Δρ_min_ = −0.32 e Å^−^^3^

### Special details

**Table:**

Experimental. The data was collected with the Oxford Cyrosystem Cobra low-temperature attachment.
Geometry. All e.s.d.'s (except the e.s.d. in the dihedral angle between two l.s. planes) are estimated using the full covariance matrix. The cell e.s.d.'s are taken into account individually in the estimation of e.s.d.'s in distances, angles and torsion angles; correlations between e.s.d.'s in cell parameters are only used when they are defined by crystal symmetry. An approximate (isotropic) treatment of cell e.s.d.'s is used for estimating e.s.d.'s involving l.s. planes.
Refinement. Refinement of *F*^2^ against ALL reflections. The weighted *R*-factor *wR* and goodness of fit *S* are based on *F*^2^, conventional *R*-factors *R* are based on *F*, with *F* set to zero for negative *F*^2^. The threshold expression of *F*^2^ > σ(*F*^2^) is used only for calculating *R*-factors(gt) *etc*. and is not relevant to the choice of reflections for refinement. *R*-factors based on *F*^2^ are statistically about twice as large as those based on *F*, and *R*- factors based on ALL data will be even larger.

### Fractional atomic coordinates and isotropic or equivalent isotropic displacement parameters (Å^2^)

**Table:**

	*x*	*y*	*z*	*U*_iso_*/*U*_eq_	
O1A	0.21415 (16)	0.83067 (9)	0.10628 (6)	0.0249 (2)	
O2A	0.19684 (16)	0.59610 (10)	0.34830 (7)	0.0292 (3)	
O3A	0.27544 (15)	0.98999 (9)	0.36114 (6)	0.0221 (2)	
O4A	0.58266 (14)	1.21472 (9)	0.13823 (6)	0.0210 (2)	
H1O4	0.597 (3)	1.164 (2)	0.1779 (13)	0.043 (6)*	
N1A	0.29206 (16)	1.11109 (10)	0.21219 (7)	0.0180 (2)	
C1A	0.23748 (19)	0.91116 (12)	0.23397 (8)	0.0174 (3)	
C2A	0.21676 (19)	0.81294 (12)	0.18822 (8)	0.0187 (3)	
C3A	0.2025 (2)	0.70672 (12)	0.22379 (9)	0.0205 (3)	
H3AA	0.1881	0.6424	0.1915	0.025*	
C4A	0.20979 (19)	0.69651 (13)	0.30743 (9)	0.0206 (3)	
C5A	0.2352 (2)	0.78998 (13)	0.35564 (9)	0.0206 (3)	
H5AA	0.2417	0.7813	0.4129	0.025*	
C6A	0.25065 (19)	0.89475 (12)	0.31884 (8)	0.0181 (3)	
C7A	0.23176 (19)	1.01974 (12)	0.19062 (8)	0.0184 (3)	
H7AA	0.1768	1.0241	0.1400	0.022*	
C8A	0.2556 (2)	1.20881 (12)	0.16002 (8)	0.0175 (3)	
C9A	0.40463 (19)	1.25893 (12)	0.12645 (8)	0.0179 (3)	
C10A	0.3708 (2)	1.35666 (13)	0.07741 (8)	0.0212 (3)	
H10A	0.4711	1.3905	0.0541	0.025*	
C11A	0.1919 (2)	1.40519 (13)	0.06215 (9)	0.0227 (3)	
H11A	0.1706	1.4718	0.0286	0.027*	
C12A	0.0448 (2)	1.35683 (13)	0.09567 (9)	0.0231 (3)	
H12A	−0.0776	1.3907	0.0857	0.028*	
C13A	0.0767 (2)	1.25830 (13)	0.14406 (9)	0.0216 (3)	
H13A	−0.0244	1.2245	0.1664	0.026*	
C14A	0.2055 (2)	0.73234 (14)	0.05636 (9)	0.0283 (4)	
H14A	0.2138	0.7546	−0.0013	0.042*	
H14B	0.0886	0.7047	0.0698	0.042*	
H14C	0.3082	0.6705	0.0664	0.042*	
C15A	0.1543 (2)	0.50165 (14)	0.30235 (12)	0.0311 (4)	
H15A	0.1412	0.4364	0.3389	0.047*	
H15B	0.2539	0.4776	0.2600	0.047*	
H15C	0.0387	0.5264	0.2767	0.047*	
C16A	0.2807 (3)	0.97923 (15)	0.44755 (9)	0.0296 (4)	
H16A	0.2969	1.0533	0.4708	0.044*	
H16B	0.3838	0.9192	0.4602	0.044*	
H16C	0.1650	0.9578	0.4710	0.044*	
O1B	0.72943 (15)	0.80124 (9)	0.39270 (6)	0.0223 (2)	
O2B	0.67530 (15)	0.56611 (9)	0.15954 (6)	0.0231 (2)	
O3B	0.73981 (16)	0.96203 (9)	0.13077 (6)	0.0249 (2)	
O4B	0.38451 (14)	1.24458 (9)	0.33850 (6)	0.0221 (2)	
H2O4	0.389 (3)	1.186 (2)	0.3050 (14)	0.053 (7)*	
N1B	0.69329 (16)	1.09095 (10)	0.27678 (7)	0.0186 (2)	
C1B	0.73499 (19)	0.88062 (12)	0.26160 (8)	0.0178 (3)	
C6B	0.72703 (19)	0.86652 (13)	0.17664 (8)	0.0187 (3)	
C5B	0.7097 (2)	0.76005 (13)	0.14376 (8)	0.0202 (3)	
H5BA	0.7063	0.7512	0.0865	0.024*	
C4B	0.69737 (19)	0.66679 (12)	0.19584 (8)	0.0182 (3)	
C3B	0.70674 (19)	0.67617 (12)	0.27957 (8)	0.0183 (3)	
H3BA	0.7005	0.6115	0.3144	0.022*	
C2B	0.72559 (19)	0.78316 (13)	0.31088 (8)	0.0176 (3)	
C7B	0.74811 (19)	0.98811 (12)	0.30235 (8)	0.0182 (3)	
H7BA	0.8028	0.9816	0.3530	0.022*	
C8B	0.71474 (19)	1.18242 (12)	0.32963 (8)	0.0175 (3)	
C9B	0.5554 (2)	1.26007 (12)	0.35589 (8)	0.0178 (3)	
C10B	0.5708 (2)	1.35514 (13)	0.40419 (8)	0.0206 (3)	
H10B	0.4644	1.4100	0.4201	0.025*	
C11B	0.7414 (2)	1.36955 (13)	0.42898 (9)	0.0246 (3)	
H11B	0.7507	1.4335	0.4627	0.030*	
C12B	0.8977 (2)	1.29117 (14)	0.40481 (9)	0.0252 (3)	
H12B	1.0138	1.3007	0.4226	0.030*	
C13B	0.8846 (2)	1.19849 (14)	0.35456 (9)	0.0219 (3)	
H13B	0.9924	1.1457	0.3371	0.026*	
C14B	0.7287 (2)	0.70361 (14)	0.44500 (9)	0.0257 (3)	
H14D	0.7280	0.7283	0.5018	0.039*	
H14E	0.8390	0.6461	0.4311	0.039*	
H14F	0.6185	0.6692	0.4380	0.039*	
C15B	0.6564 (2)	0.47040 (13)	0.21263 (9)	0.0249 (3)	
H15D	0.6355	0.4043	0.1805	0.037*	
H15E	0.5516	0.4926	0.2528	0.037*	
H15F	0.7691	0.4488	0.2409	0.037*	
C16B	0.7402 (3)	0.95075 (15)	0.04466 (9)	0.0343 (4)	
H16D	0.7459	1.0261	0.0190	0.051*	
H16E	0.6274	0.9236	0.0312	0.051*	
H16F	0.8477	0.8947	0.0245	0.051*	

### Atomic displacement parameters (Å^2^)

**Table:**

	*U*^11^	*U*^22^	*U*^33^	*U*^12^	*U*^13^	*U*^23^
O1A	0.0414 (6)	0.0140 (5)	0.0205 (5)	−0.0081 (5)	−0.0030 (4)	−0.0023 (4)
O2A	0.0367 (6)	0.0142 (5)	0.0373 (6)	−0.0064 (5)	−0.0023 (5)	0.0087 (5)
O3A	0.0320 (6)	0.0150 (5)	0.0194 (5)	−0.0032 (4)	−0.0044 (4)	−0.0008 (4)
O4A	0.0235 (5)	0.0143 (5)	0.0247 (5)	−0.0016 (4)	−0.0030 (4)	0.0060 (4)
N1A	0.0231 (6)	0.0106 (6)	0.0204 (5)	−0.0027 (5)	−0.0029 (4)	0.0024 (4)
C1A	0.0199 (6)	0.0114 (6)	0.0209 (6)	−0.0033 (5)	−0.0005 (5)	0.0003 (5)
C2A	0.0215 (7)	0.0139 (7)	0.0206 (6)	−0.0032 (5)	−0.0008 (5)	−0.0006 (5)
C3A	0.0216 (7)	0.0107 (7)	0.0291 (7)	−0.0023 (5)	−0.0009 (5)	0.0000 (5)
C4A	0.0183 (6)	0.0122 (7)	0.0306 (7)	−0.0015 (5)	0.0004 (5)	0.0058 (5)
C5A	0.0219 (7)	0.0172 (7)	0.0217 (6)	−0.0004 (6)	−0.0011 (5)	0.0044 (5)
C6A	0.0177 (6)	0.0134 (7)	0.0225 (6)	−0.0007 (5)	−0.0010 (5)	−0.0006 (5)
C7A	0.0229 (7)	0.0135 (7)	0.0187 (6)	−0.0026 (5)	−0.0013 (5)	−0.0005 (5)
C8A	0.0265 (7)	0.0102 (6)	0.0159 (6)	−0.0028 (5)	−0.0032 (5)	0.0002 (5)
C9A	0.0245 (7)	0.0102 (6)	0.0186 (6)	−0.0006 (5)	−0.0033 (5)	−0.0010 (5)
C10A	0.0307 (7)	0.0126 (7)	0.0204 (6)	−0.0044 (6)	−0.0022 (5)	0.0024 (5)
C11A	0.0354 (8)	0.0124 (7)	0.0197 (6)	−0.0002 (6)	−0.0058 (6)	0.0028 (5)
C12A	0.0281 (7)	0.0180 (7)	0.0217 (7)	0.0029 (6)	−0.0065 (5)	0.0003 (5)
C13A	0.0252 (7)	0.0178 (7)	0.0214 (6)	−0.0023 (6)	−0.0030 (5)	−0.0003 (5)
C14A	0.0417 (9)	0.0196 (8)	0.0251 (7)	−0.0108 (7)	0.0011 (6)	−0.0073 (6)
C15A	0.0290 (8)	0.0124 (7)	0.0527 (10)	−0.0054 (6)	−0.0043 (7)	0.0068 (7)
C16A	0.0450 (9)	0.0232 (8)	0.0199 (7)	−0.0011 (7)	−0.0070 (6)	−0.0005 (6)
O1B	0.0351 (6)	0.0147 (5)	0.0166 (5)	−0.0032 (4)	−0.0006 (4)	0.0012 (4)
O2B	0.0333 (6)	0.0137 (5)	0.0229 (5)	−0.0053 (4)	−0.0017 (4)	−0.0016 (4)
O3B	0.0439 (6)	0.0113 (5)	0.0179 (5)	−0.0004 (5)	−0.0014 (4)	0.0026 (4)
O4B	0.0219 (5)	0.0165 (5)	0.0274 (5)	−0.0010 (4)	−0.0028 (4)	−0.0063 (4)
N1B	0.0225 (6)	0.0125 (6)	0.0197 (5)	0.0002 (5)	−0.0013 (4)	−0.0018 (4)
C1B	0.0196 (6)	0.0121 (7)	0.0204 (6)	0.0005 (5)	−0.0006 (5)	0.0005 (5)
C6B	0.0211 (6)	0.0139 (7)	0.0195 (6)	0.0010 (5)	−0.0004 (5)	0.0011 (5)
C5B	0.0253 (7)	0.0155 (7)	0.0182 (6)	0.0012 (6)	−0.0014 (5)	−0.0007 (5)
C4B	0.0187 (6)	0.0132 (7)	0.0219 (6)	−0.0006 (5)	−0.0002 (5)	−0.0016 (5)
C3B	0.0204 (6)	0.0123 (6)	0.0214 (6)	−0.0012 (5)	0.0008 (5)	0.0013 (5)
C2B	0.0183 (6)	0.0158 (7)	0.0174 (6)	0.0007 (5)	0.0003 (5)	−0.0003 (5)
C7B	0.0217 (6)	0.0143 (7)	0.0181 (6)	−0.0011 (5)	−0.0010 (5)	0.0000 (5)
C8B	0.0242 (7)	0.0118 (6)	0.0162 (6)	−0.0017 (5)	−0.0020 (5)	0.0014 (5)
C9B	0.0241 (7)	0.0121 (6)	0.0170 (6)	−0.0026 (5)	−0.0019 (5)	0.0015 (5)
C10B	0.0304 (7)	0.0128 (7)	0.0181 (6)	−0.0033 (6)	0.0007 (5)	0.0004 (5)
C11B	0.0374 (8)	0.0169 (7)	0.0218 (7)	−0.0106 (6)	−0.0037 (6)	−0.0002 (5)
C12B	0.0300 (8)	0.0232 (8)	0.0250 (7)	−0.0103 (7)	−0.0070 (6)	0.0045 (6)
C13B	0.0237 (7)	0.0182 (7)	0.0236 (7)	−0.0034 (6)	−0.0020 (5)	0.0037 (5)
C14B	0.0392 (9)	0.0195 (8)	0.0185 (7)	−0.0050 (7)	−0.0030 (6)	0.0042 (6)
C15B	0.0310 (8)	0.0165 (7)	0.0288 (7)	−0.0086 (6)	−0.0019 (6)	0.0007 (6)
C16B	0.0635 (12)	0.0194 (8)	0.0185 (7)	−0.0026 (8)	−0.0030 (7)	0.0031 (6)

### Geometric parameters (Å, º)

**Table:**

O1A—C2A	1.3620 (17)	O1B—C2B	1.3665 (16)
O1A—C14A	1.4324 (16)	O1B—C14B	1.4240 (18)
O2A—C4A	1.3616 (18)	O2B—C4B	1.3647 (16)
O2A—C15A	1.4345 (19)	O2B—C15B	1.4321 (18)
O3A—C6A	1.3641 (16)	O3B—C6B	1.3547 (17)
O3A—C16A	1.4276 (17)	O3B—C16B	1.4225 (17)
O4A—C9A	1.3579 (17)	O4B—C9B	1.3555 (17)
O4A—H1O4	0.88 (2)	O4B—H2O4	0.87 (2)
N1A—C7A	1.2868 (17)	N1B—C7B	1.2823 (19)
N1A—C8A	1.4251 (18)	N1B—C8B	1.4213 (17)
C1A—C6A	1.4149 (19)	C1B—C2B	1.401 (2)
C1A—C2A	1.4149 (18)	C1B—C6B	1.4145 (19)
C1A—C7A	1.447 (2)	C1B—C7B	1.4514 (19)
C2A—C3A	1.385 (2)	C6B—C5B	1.3918 (19)
C3A—C4A	1.383 (2)	C5B—C4B	1.392 (2)
C3A—H3AA	0.9500	C5B—H5BA	0.9500
C4A—C5A	1.402 (2)	C4B—C3B	1.3894 (19)
C5A—C6A	1.380 (2)	C3B—C2B	1.3903 (19)
C5A—H5AA	0.9500	C3B—H3BA	0.9500
C7A—H7AA	0.9500	C7B—H7BA	0.9500
C8A—C13A	1.395 (2)	C8B—C13B	1.390 (2)
C8A—C9A	1.405 (2)	C8B—C9B	1.4040 (19)
C9A—C10A	1.393 (2)	C9B—C10B	1.3977 (18)
C10A—C11A	1.388 (2)	C10B—C11B	1.389 (2)
C10A—H10A	0.9500	C10B—H10B	0.9500
C11A—C12A	1.382 (2)	C11B—C12B	1.385 (2)
C11A—H11A	0.9500	C11B—H11B	0.9500
C12A—C13A	1.393 (2)	C12B—C13B	1.390 (2)
C12A—H12A	0.9500	C12B—H12B	0.9500
C13A—H13A	0.9500	C13B—H13B	0.9500
C14A—H14A	0.9800	C14B—H14D	0.9800
C14A—H14B	0.9800	C14B—H14E	0.9800
C14A—H14C	0.9800	C14B—H14F	0.9800
C15A—H15A	0.9800	C15B—H15D	0.9800
C15A—H15B	0.9800	C15B—H15E	0.9800
C15A—H15C	0.9800	C15B—H15F	0.9800
C16A—H16A	0.9800	C16B—H16D	0.9800
C16A—H16B	0.9800	C16B—H16E	0.9800
C16A—H16C	0.9800	C16B—H16F	0.9800
			
C2A—O1A—C14A	117.14 (12)	C2B—O1B—C14B	117.47 (11)
C4A—O2A—C15A	117.14 (12)	C4B—O2B—C15B	116.49 (11)
C6A—O3A—C16A	117.32 (12)	C6B—O3B—C16B	117.65 (12)
C9A—O4A—H1O4	114.6 (14)	C9B—O4B—H2O4	111.6 (15)
C7A—N1A—C8A	115.68 (12)	C7B—N1B—C8B	115.77 (12)
C6A—C1A—C2A	116.46 (13)	C2B—C1B—C6B	117.29 (13)
C6A—C1A—C7A	126.30 (12)	C2B—C1B—C7B	117.12 (12)
C2A—C1A—C7A	117.12 (12)	C6B—C1B—C7B	125.57 (13)
O1A—C2A—C3A	122.60 (12)	O3B—C6B—C5B	123.26 (12)
O1A—C2A—C1A	114.70 (12)	O3B—C6B—C1B	115.65 (12)
C3A—C2A—C1A	122.69 (13)	C5B—C6B—C1B	121.09 (14)
C4A—C3A—C2A	118.44 (13)	C4B—C5B—C6B	119.10 (13)
C4A—C3A—H3AA	120.8	C4B—C5B—H5BA	120.4
C2A—C3A—H3AA	120.8	C6B—C5B—H5BA	120.4
O2A—C4A—C3A	123.05 (13)	O2B—C4B—C3B	122.34 (13)
O2A—C4A—C5A	115.53 (13)	O2B—C4B—C5B	115.84 (12)
C3A—C4A—C5A	121.40 (13)	C3B—C4B—C5B	121.83 (13)
C6A—C5A—C4A	119.17 (13)	C4B—C3B—C2B	117.98 (13)
C6A—C5A—H5AA	120.4	C4B—C3B—H3BA	121.0
C4A—C5A—H5AA	120.4	C2B—C3B—H3BA	121.0
O3A—C6A—C5A	122.97 (12)	O1B—C2B—C3B	121.90 (13)
O3A—C6A—C1A	115.27 (12)	O1B—C2B—C1B	115.37 (12)
C5A—C6A—C1A	121.75 (12)	C3B—C2B—C1B	122.69 (12)
N1A—C7A—C1A	127.95 (13)	N1B—C7B—C1B	126.50 (13)
N1A—C7A—H7AA	116.0	N1B—C7B—H7BA	116.7
C1A—C7A—H7AA	116.0	C1B—C7B—H7BA	116.7
C13A—C8A—C9A	119.40 (13)	C13B—C8B—C9B	119.66 (13)
C13A—C8A—N1A	121.91 (13)	C13B—C8B—N1B	123.03 (13)
C9A—C8A—N1A	118.64 (13)	C9B—C8B—N1B	117.30 (12)
O4A—C9A—C10A	117.89 (13)	O4B—C9B—C10B	118.09 (12)
O4A—C9A—C8A	122.80 (13)	O4B—C9B—C8B	122.48 (12)
C10A—C9A—C8A	119.30 (13)	C10B—C9B—C8B	119.40 (13)
C11A—C10A—C9A	120.70 (14)	C11B—C10B—C9B	120.17 (14)
C11A—C10A—H10A	119.6	C11B—C10B—H10B	119.9
C9A—C10A—H10A	119.6	C9B—C10B—H10B	119.9
C12A—C11A—C10A	120.20 (14)	C12B—C11B—C10B	120.32 (13)
C12A—C11A—H11A	119.9	C12B—C11B—H11B	119.8
C10A—C11A—H11A	119.9	C10B—C11B—H11B	119.8
C11A—C12A—C13A	119.72 (14)	C11B—C12B—C13B	119.90 (14)
C11A—C12A—H12A	120.1	C11B—C12B—H12B	120.1
C13A—C12A—H12A	120.1	C13B—C12B—H12B	120.1
C12A—C13A—C8A	120.66 (14)	C12B—C13B—C8B	120.49 (14)
C12A—C13A—H13A	119.7	C12B—C13B—H13B	119.8
C8A—C13A—H13A	119.7	C8B—C13B—H13B	119.8
O1A—C14A—H14A	109.5	O1B—C14B—H14D	109.5
O1A—C14A—H14B	109.5	O1B—C14B—H14E	109.5
H14A—C14A—H14B	109.5	H14D—C14B—H14E	109.5
O1A—C14A—H14C	109.5	O1B—C14B—H14F	109.5
H14A—C14A—H14C	109.5	H14D—C14B—H14F	109.5
H14B—C14A—H14C	109.5	H14E—C14B—H14F	109.5
O2A—C15A—H15A	109.5	O2B—C15B—H15D	109.5
O2A—C15A—H15B	109.5	O2B—C15B—H15E	109.5
H15A—C15A—H15B	109.5	H15D—C15B—H15E	109.5
O2A—C15A—H15C	109.5	O2B—C15B—H15F	109.5
H15A—C15A—H15C	109.5	H15D—C15B—H15F	109.5
H15B—C15A—H15C	109.5	H15E—C15B—H15F	109.5
O3A—C16A—H16A	109.5	O3B—C16B—H16D	109.5
O3A—C16A—H16B	109.5	O3B—C16B—H16E	109.5
H16A—C16A—H16B	109.5	H16D—C16B—H16E	109.5
O3A—C16A—H16C	109.5	O3B—C16B—H16F	109.5
H16A—C16A—H16C	109.5	H16D—C16B—H16F	109.5
H16B—C16A—H16C	109.5	H16E—C16B—H16F	109.5
			
C14A—O1A—C2A—C3A	−3.2 (2)	C16B—O3B—C6B—C5B	−1.7 (2)
C14A—O1A—C2A—C1A	176.06 (13)	C16B—O3B—C6B—C1B	177.43 (14)
C6A—C1A—C2A—O1A	−176.76 (12)	C2B—C1B—C6B—O3B	−178.87 (12)
C7A—C1A—C2A—O1A	6.93 (18)	C7B—C1B—C6B—O3B	2.5 (2)
C6A—C1A—C2A—C3A	2.5 (2)	C2B—C1B—C6B—C5B	0.3 (2)
C7A—C1A—C2A—C3A	−173.82 (13)	C7B—C1B—C6B—C5B	−178.30 (13)
O1A—C2A—C3A—C4A	178.87 (13)	O3B—C6B—C5B—C4B	−179.96 (13)
C1A—C2A—C3A—C4A	−0.3 (2)	C1B—C6B—C5B—C4B	0.9 (2)
C15A—O2A—C4A—C3A	−6.7 (2)	C15B—O2B—C4B—C3B	1.86 (19)
C15A—O2A—C4A—C5A	174.54 (13)	C15B—O2B—C4B—C5B	−178.03 (12)
C2A—C3A—C4A—O2A	179.90 (13)	C6B—C5B—C4B—O2B	178.19 (12)
C2A—C3A—C4A—C5A	−1.5 (2)	C6B—C5B—C4B—C3B	−1.7 (2)
O2A—C4A—C5A—C6A	179.65 (12)	O2B—C4B—C3B—C2B	−178.70 (12)
C3A—C4A—C5A—C6A	0.9 (2)	C5B—C4B—C3B—C2B	1.2 (2)
C16A—O3A—C6A—C5A	−1.5 (2)	C14B—O1B—C2B—C3B	5.07 (19)
C16A—O3A—C6A—C1A	177.08 (13)	C14B—O1B—C2B—C1B	−177.21 (12)
C4A—C5A—C6A—O3A	179.96 (13)	C4B—C3B—C2B—O1B	177.67 (13)
C4A—C5A—C6A—C1A	1.4 (2)	C4B—C3B—C2B—C1B	0.1 (2)
C2A—C1A—C6A—O3A	178.33 (12)	C6B—C1B—C2B—O1B	−178.54 (12)
C7A—C1A—C6A—O3A	−5.7 (2)	C7B—C1B—C2B—O1B	0.18 (18)
C2A—C1A—C6A—C5A	−3.0 (2)	C6B—C1B—C2B—C3B	−0.8 (2)
C7A—C1A—C6A—C5A	172.89 (14)	C7B—C1B—C2B—C3B	177.88 (13)
C8A—N1A—C7A—C1A	−175.58 (13)	C8B—N1B—C7B—C1B	177.48 (13)
C6A—C1A—C7A—N1A	23.4 (2)	C2B—C1B—C7B—N1B	−152.97 (14)
C2A—C1A—C7A—N1A	−160.71 (14)	C6B—C1B—C7B—N1B	25.6 (2)
C7A—N1A—C8A—C13A	58.87 (17)	C7B—N1B—C8B—C13B	59.30 (19)
C7A—N1A—C8A—C9A	−123.60 (14)	C7B—N1B—C8B—C9B	−121.81 (14)
C13A—C8A—C9A—O4A	−178.98 (12)	C13B—C8B—C9B—O4B	−175.24 (13)
N1A—C8A—C9A—O4A	3.44 (19)	N1B—C8B—C9B—O4B	5.8 (2)
C13A—C8A—C9A—C10A	−0.43 (19)	C13B—C8B—C9B—C10B	2.5 (2)
N1A—C8A—C9A—C10A	−178.01 (12)	N1B—C8B—C9B—C10B	−176.43 (12)
O4A—C9A—C10A—C11A	179.19 (12)	O4B—C9B—C10B—C11B	174.92 (13)
C8A—C9A—C10A—C11A	0.6 (2)	C8B—C9B—C10B—C11B	−2.9 (2)
C9A—C10A—C11A—C12A	0.0 (2)	C9B—C10B—C11B—C12B	1.2 (2)
C10A—C11A—C12A—C13A	−0.8 (2)	C10B—C11B—C12B—C13B	0.9 (2)
C11A—C12A—C13A—C8A	0.9 (2)	C11B—C12B—C13B—C8B	−1.3 (2)
C9A—C8A—C13A—C12A	−0.3 (2)	C9B—C8B—C13B—C12B	−0.4 (2)
N1A—C8A—C13A—C12A	177.19 (12)	N1B—C8B—C13B—C12B	178.47 (13)

### Hydrogen-bond geometry (Å, º)

**Table:**

*D*—H···*A*	*D*—H	H···*A*	*D*···*A*	*D*—H···*A*
O4*A*—H1*O*4···O3*B*	0.88 (2)	2.53 (2)	2.9924 (15)	114.0 (17)
O4*A*—H1*O*4···N1*B*	0.88 (2)	1.96 (2)	2.7897 (15)	158 (2)
O4*B*—H2*O*4···N1*A*	0.88 (2)	2.00 (2)	2.8013 (16)	151 (2)
O4*B*—H2*O*4···N1*B*	0.88 (2)	2.35 (2)	2.7891 (16)	111.1 (18)
C13*B*—H13*B*···O3*A*^i^	0.95	2.59	3.4639 (19)	154
C15*B*—H15*D*···O4*A*^ii^	0.98	2.43	3.3847 (18)	164

Symmetry codes: (i) *x*+1, *y*, *z*; (ii) *x*, *y*−1, *z*.
